# Supplementary material for: General Anesthesia Compared to Spinal Anesthesia for Patients Undergoing Lumbar Vertebral Surgery: A Meta-Analysis of Randomized Controlled Trials
Source: J Clin Med. 2020 Dec 30;10(1):102. doi: 10.3390/jcm10010102 (PMC7796239; doi:10.3390/jcm10010102)
Supplement: Supplementary file 1 [file jcm-10-00102-s001.zip › Suppl/Figure S1.docx]

**PostHoc) Pain after 24 hours**

**
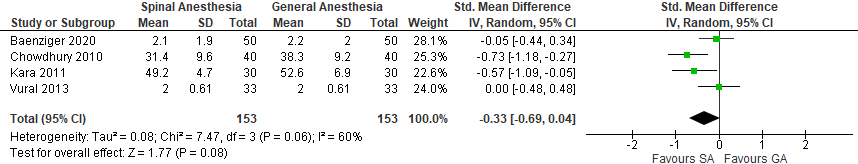
**

**a) Analgesic Requirement**


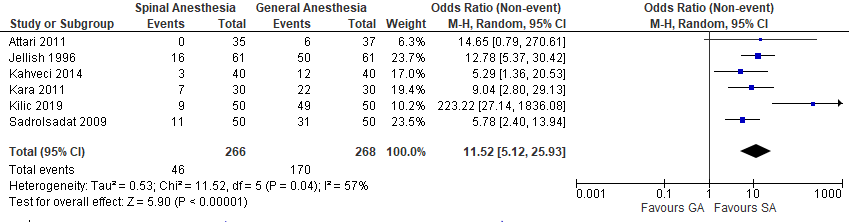


**b) Blood Loss**


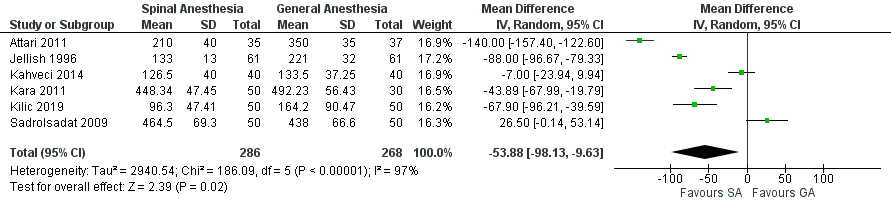


**c) Surgery Length**


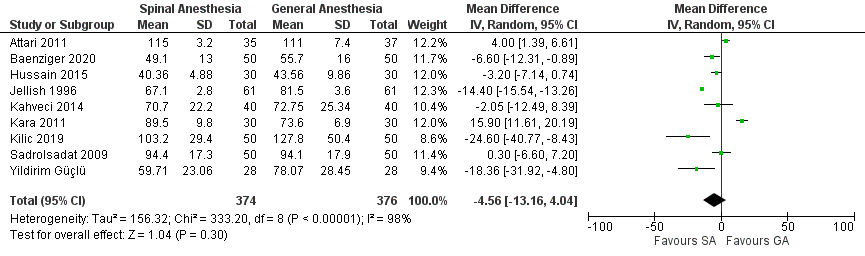


**d) Hypotension**


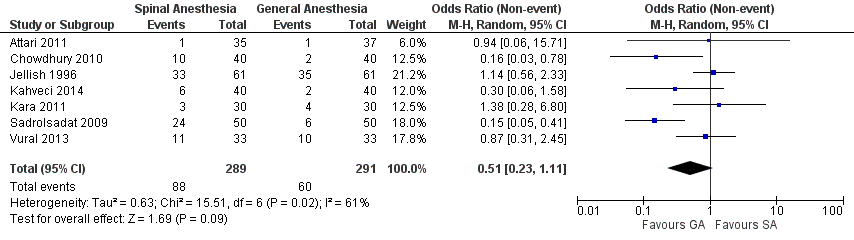


- Continuity correction for Attari’s study

**d) Bradycardia**


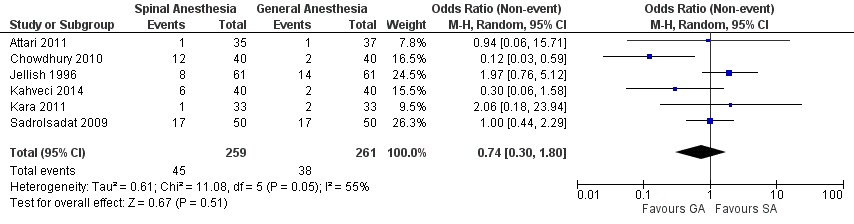


- Continuity correction for Attari’s study

**e) Nausea and Vomiting**


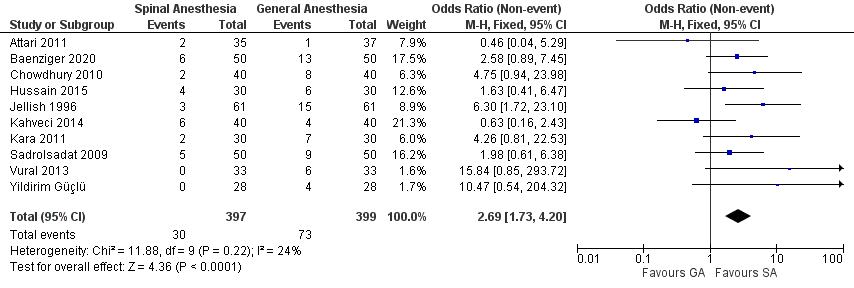


**f) Urinary Retention**


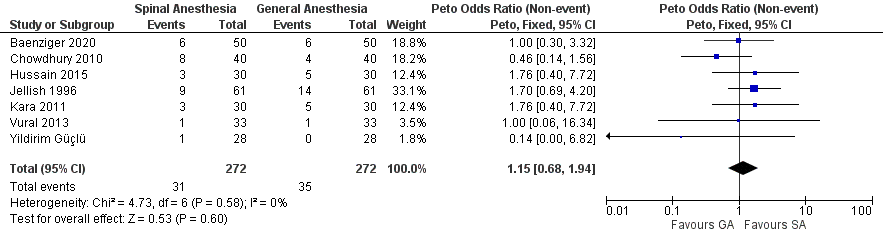


- Continuity correction for Vural’s study

**g) Length of Stay**


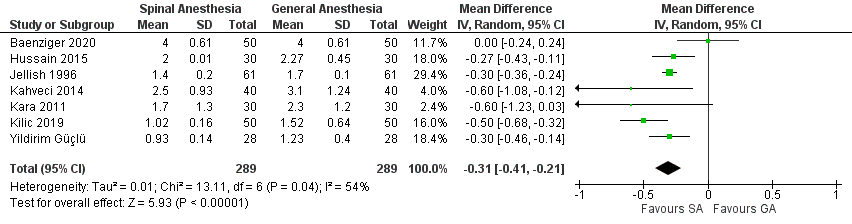


**h) Patient Satisfaction**


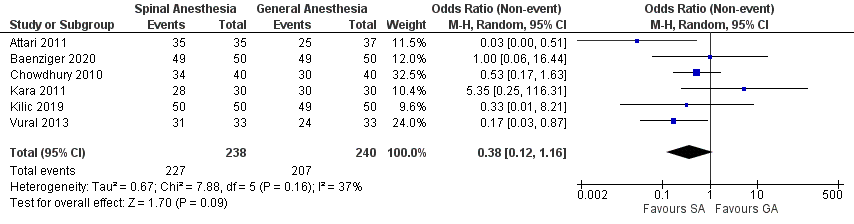


- Continuity correction for Baezinger’s study

**h) Surgeon Satisfaction**


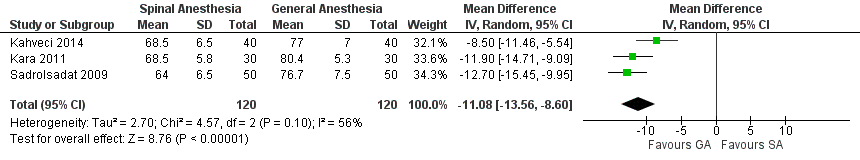


Figure S1. Secondary outcomes forest plots.
