# Supplementary material for: General Anesthesia Compared to Spinal Anesthesia for Patients Undergoing Lumbar Vertebral Surgery: A Meta-Analysis of Randomized Controlled Trials
Source: J Clin Med. 2020 Dec 30;10(1):102. doi: 10.3390/jcm10010102 (PMC7796239; doi:10.3390/jcm10010102)
Supplement: Supplementary file 1 [file jcm-10-00102-s001.zip › Suppl/Figure S2.docx]

**a) Analgesic Requirement**

**
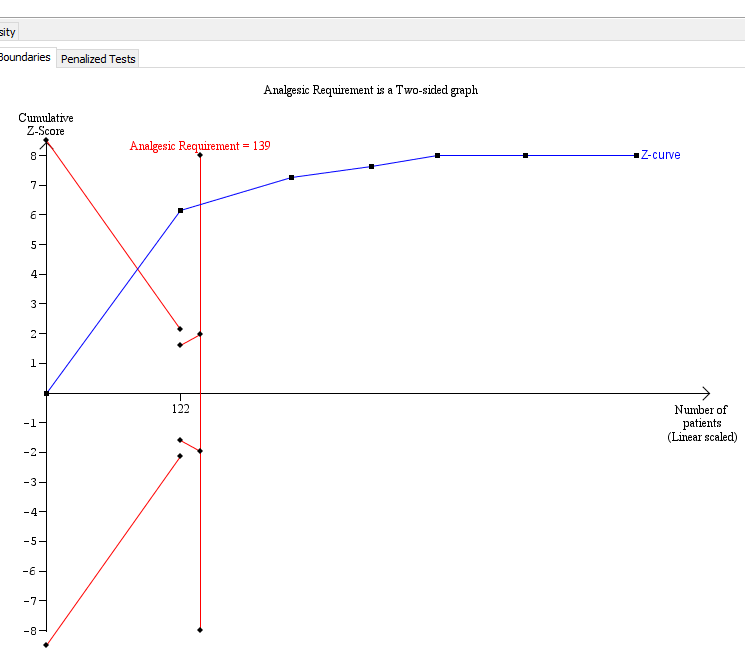
**

**b) Blood Loss**

**
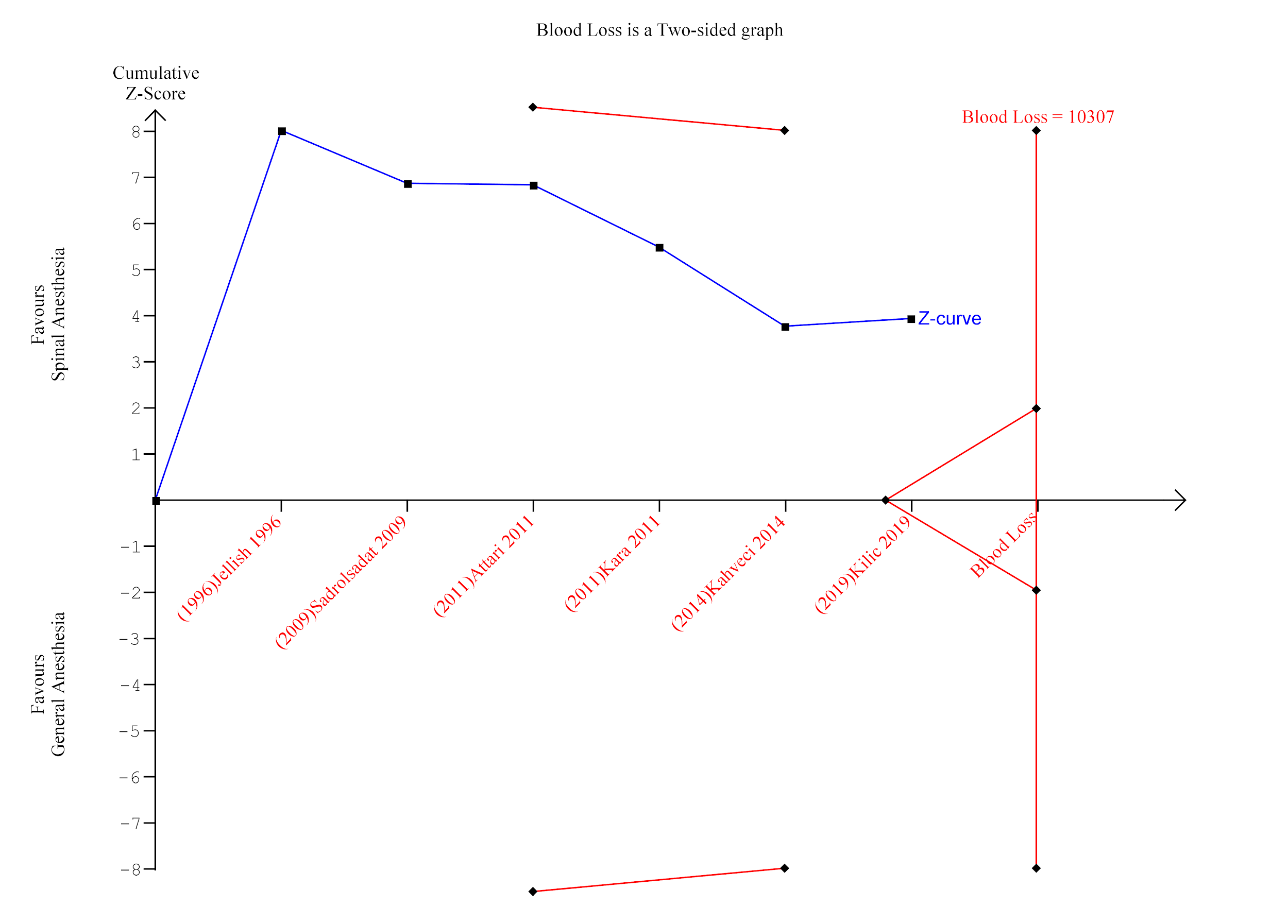
**

**c) Surgery Length**

**
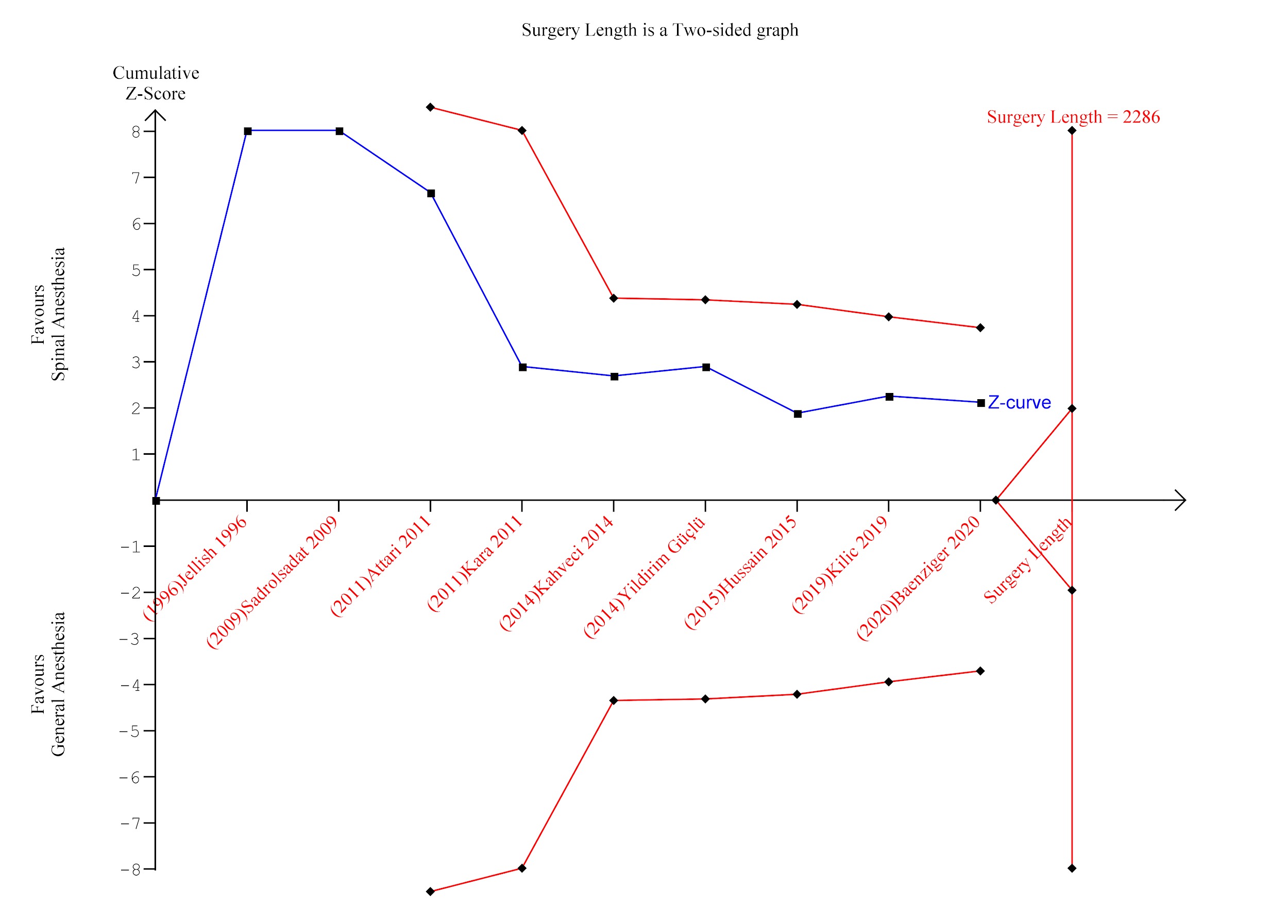
**

**d) Hypotension**

**
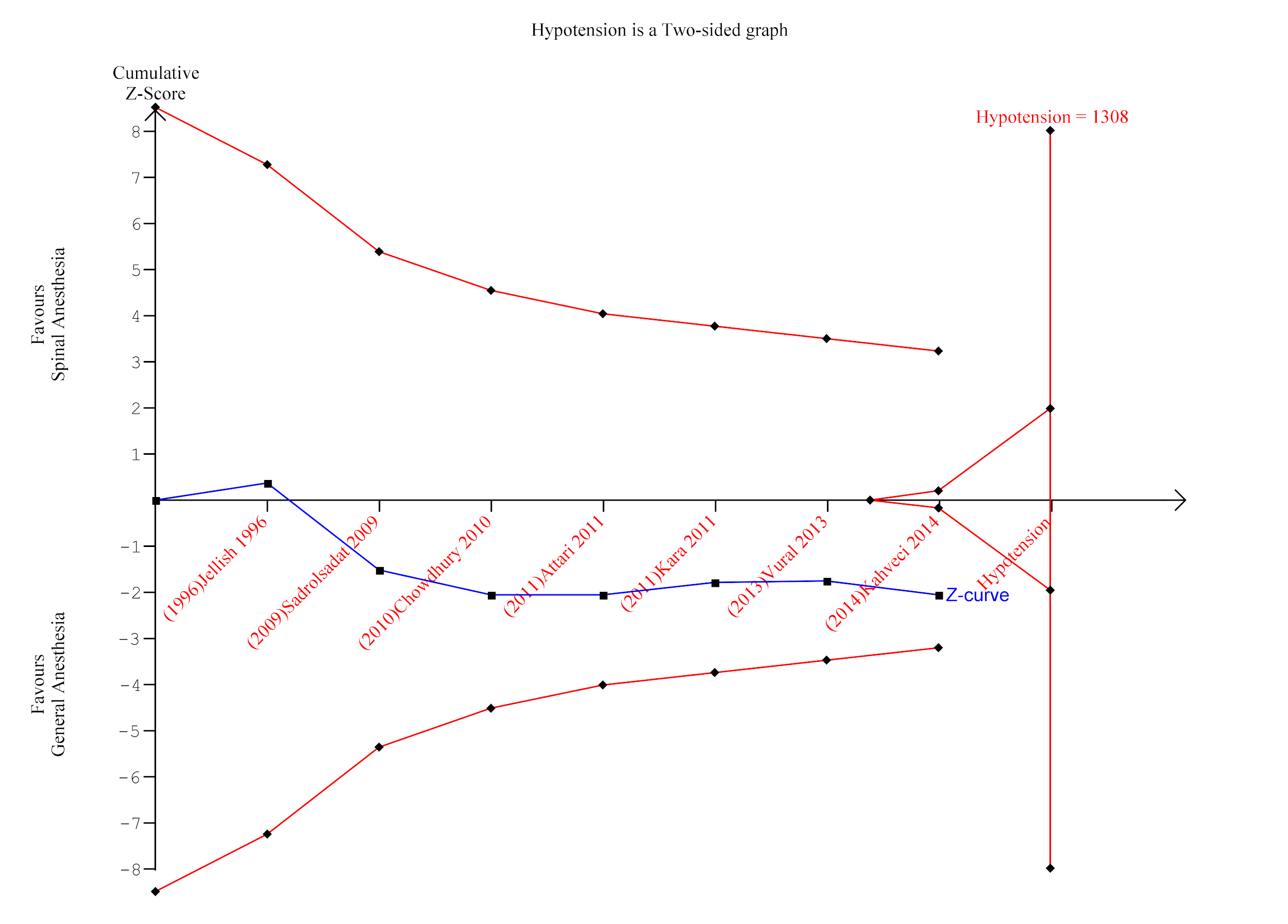
**

**d) Bradycardia**

**
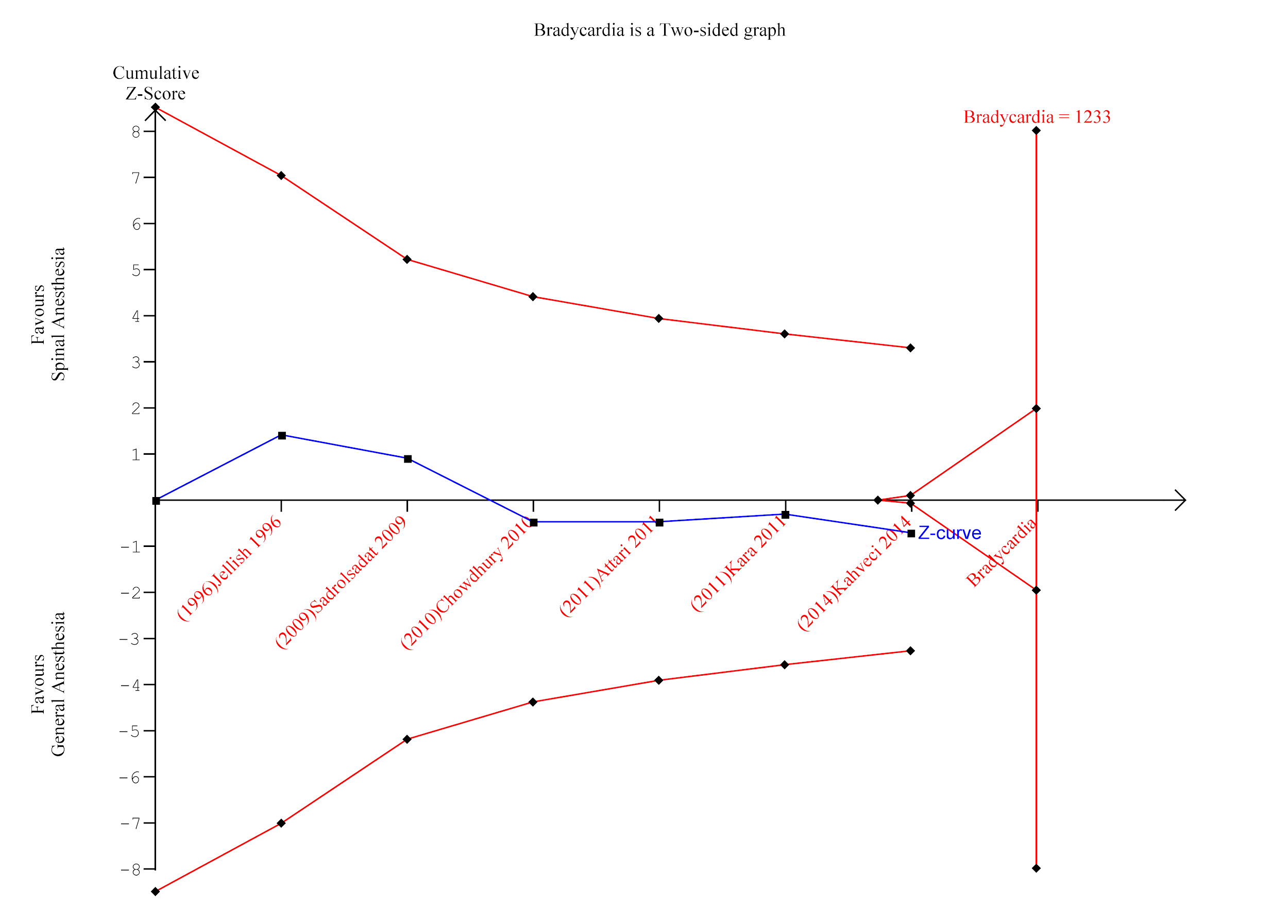
**

**e) Nausea and Vomiting**

**
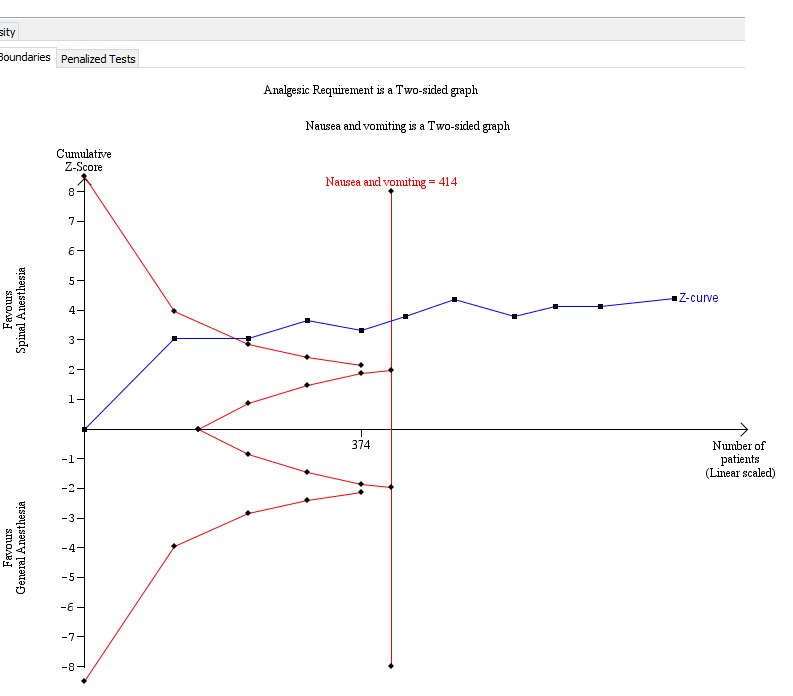
**

**f) Urinary Retention**

**
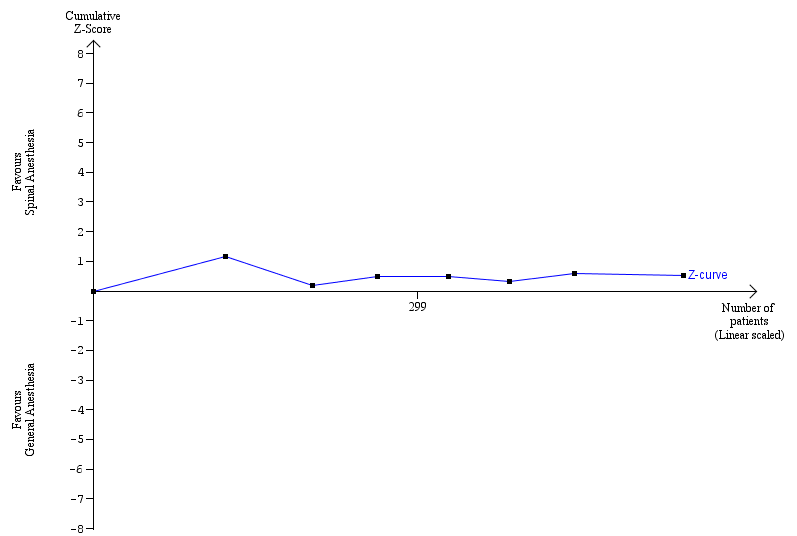
**

**g) Length of Stay**

**
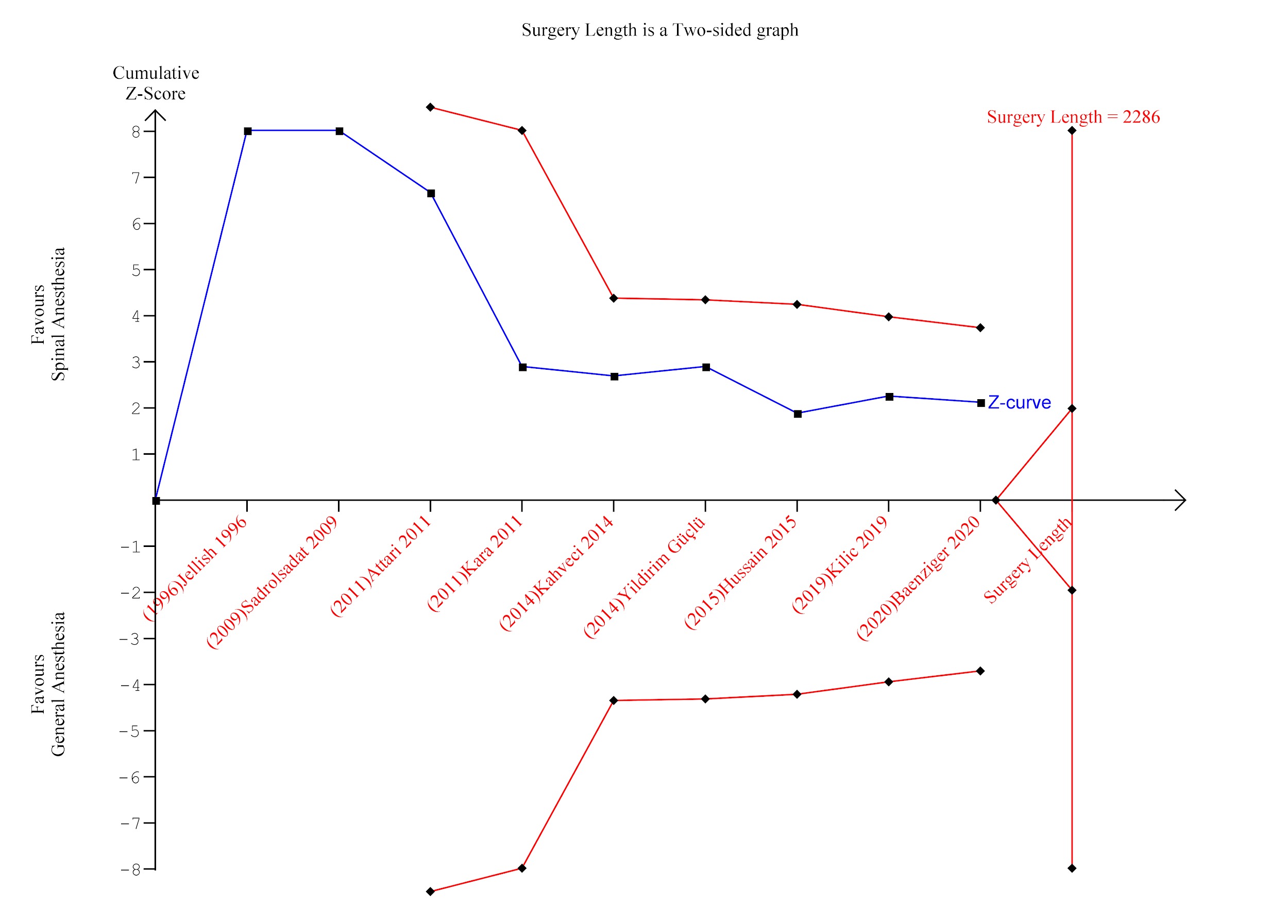
**

**h) Patient Satisfaction**

**
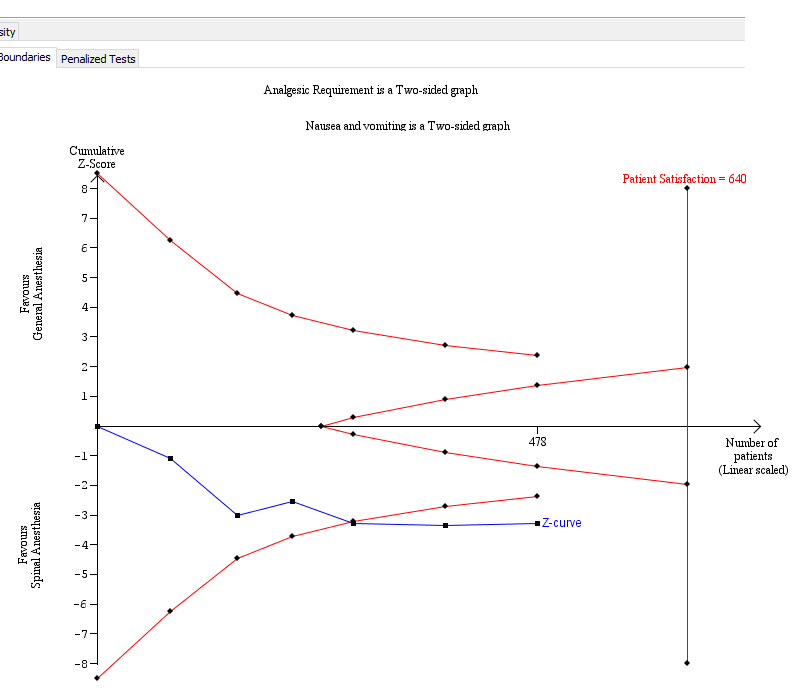
**

**h) Surgeon Satisfaction**

**
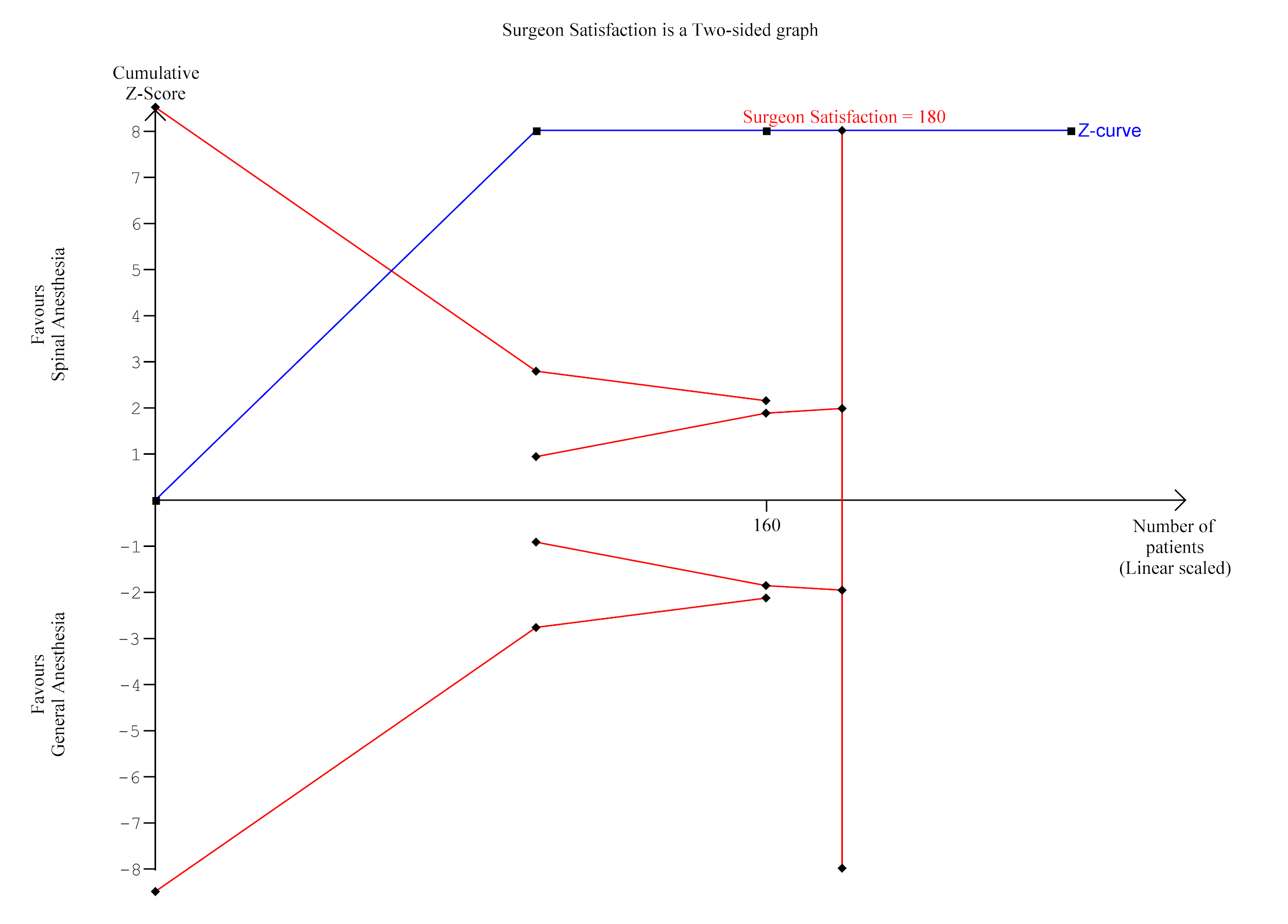
**

Figure S2. Trial sequence analysis.
