# Supplementary material for: General Anesthesia Compared to Spinal Anesthesia for Patients Undergoing Lumbar Vertebral Surgery: A Meta-Analysis of Randomized Controlled Trials
Source: J Clin Med. 2020 Dec 30;10(1):102. doi: 10.3390/jcm10010102 (PMC7796239; doi:10.3390/jcm10010102)
Supplement: Supplementary file 1 [file jcm-10-00102-s001.zip › Suppl/Figure S3.docx]

**Postoperative Pain**

**
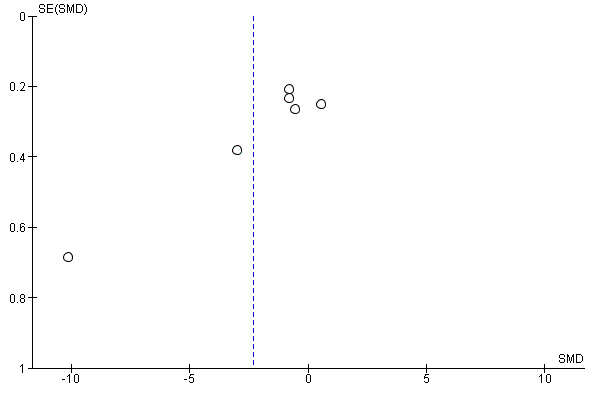
**

**PostHoc) Pain after 24 hours**

**
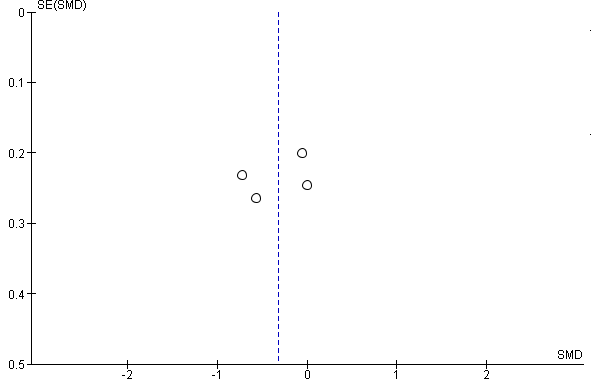
**

**a)Analgesic Requirement**


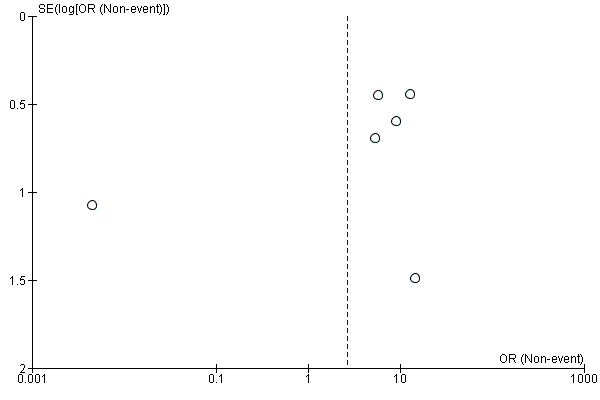


**b)Blood Loss**


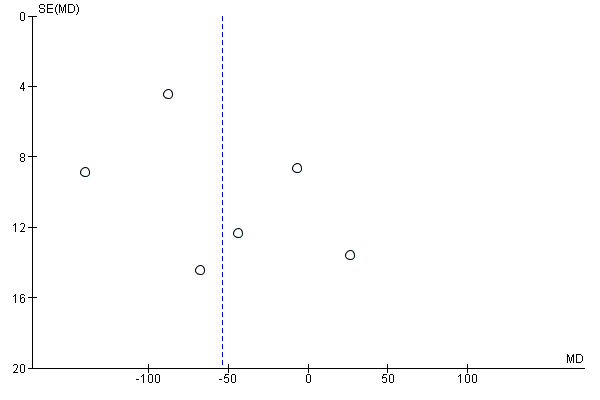


**c)Surgery Length**


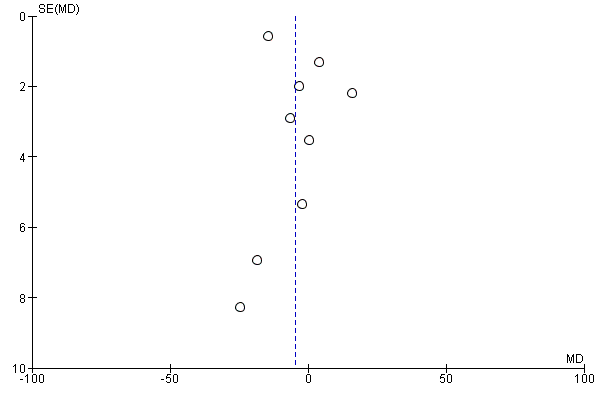


**d)Hypotension**


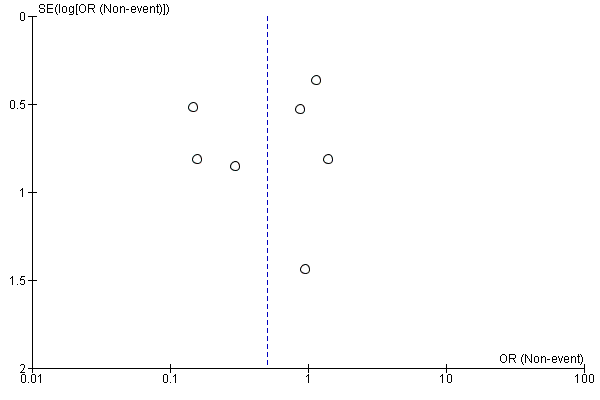


**d)Bradycardia**


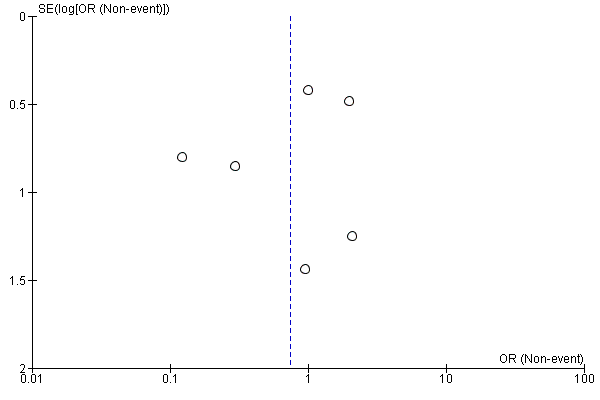


**e)Nausea and Vomiting**


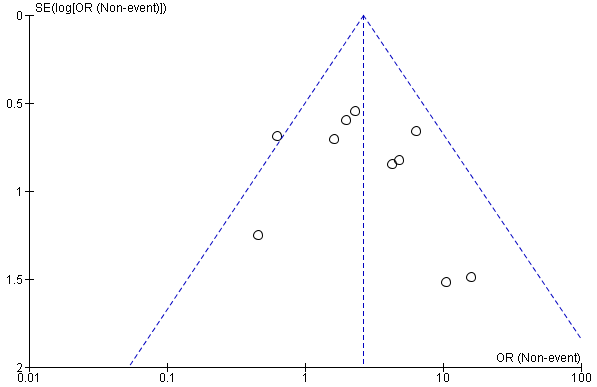


**f)Urinary Retention**


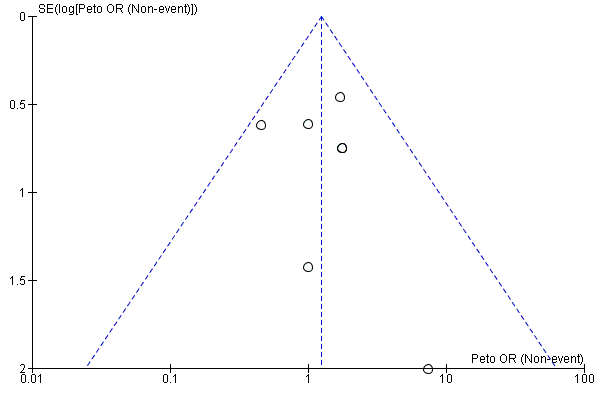


**g)Length of Stay**


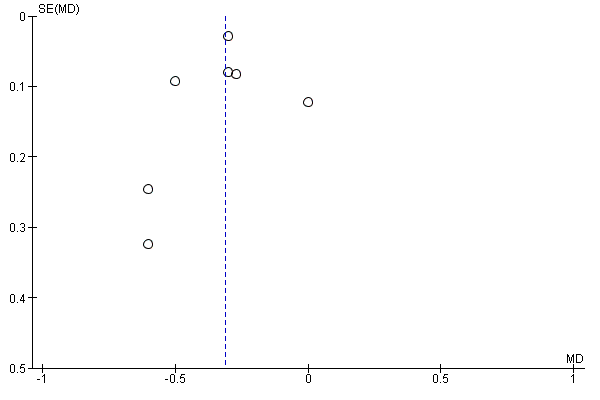


**h) Patient Satisfaction**


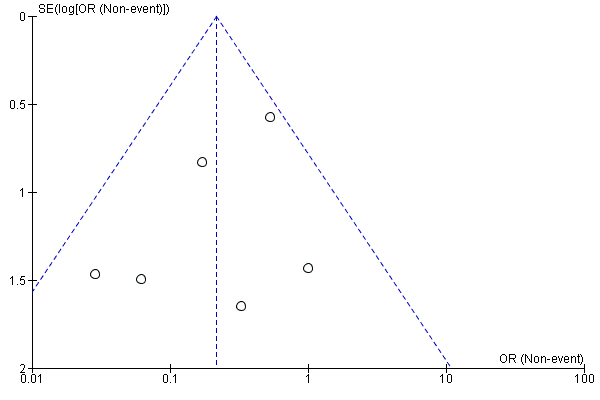


**h)Surgeon Satisfaction**


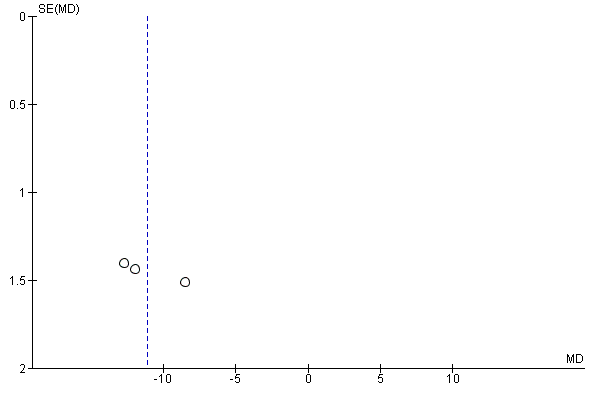


Figure S3. Funnel plots.
