# Supplementary material for: General Anesthesia Compared to Spinal Anesthesia for Patients Undergoing Lumbar Vertebral Surgery: A Meta-Analysis of Randomized Controlled Trials
Source: J Clin Med. 2020 Dec 30;10(1):102. doi: 10.3390/jcm10010102 (PMC7796239; doi:10.3390/jcm10010102)
Supplement: Supplementary file 1 [file jcm-10-00102-s001.zip › Suppl/Table S1.docx]

Table S1. Search strategy.

| PUBMED | (“Anesthesia, Spinal”[Mesh] OR "spinal anesthesia" OR “spinal anaesthesia”) and (“Anesthesia, General”[Mesh] OR "general anesthesia" OR “general anaesthesia”) and (vertebral or lumbar or laminectomy or discectomy) |
| --- | --- |
| GOOGLE Scholar | ("spinal anesthesia" OR “spinal anaesthesia”) AND (“general anesthesia" OR “general anaesthesia”) AND (vertebral or lumbar or laminectomy or discectomy) |
| CENTRAL, the  Cochrane Library | ("spinal anesthesia" OR “spinal anaesthesia”) AND (“general anesthesia" OR “general anaesthesia”) AND (vertebral or lumbar or laminectomy or discectomy) |
