# Supplementary material for: General Anesthesia Compared to Spinal Anesthesia for Patients Undergoing Lumbar Vertebral Surgery: A Meta-Analysis of Randomized Controlled Trials
Source: J Clin Med. 2020 Dec 30;10(1):102. doi: 10.3390/jcm10010102 (PMC7796239; doi:10.3390/jcm10010102)
Supplement: Supplementary file 1 [file jcm-10-00102-s001.zip › Suppl/Table S2.docx]

Table S2. Group comparison.

| Study | Age | | Gender (F) | | ASA-PS(I/II/III) | | Weight(Kg )/ BMI(Kg/cm^2^) | |
| --- | --- | --- | --- | --- | --- | --- | --- | --- |
|  | SA | GA | SA | GA | SA | GA | SA | GA |
| Attari (2001) | 42.1±3.1 | 45.1±2.9 | 13(37.14%) | 20(54.05%) | 11/24/0 | 15/22/0 | 75±4.0 / NR | 72±3.8 / NR |
| Baenziger (2020) | 62.5 (IQR 44.8-  75.3) | 61.0 (48.8;  71.0) | 21 (42.00%) | 25 (50.00%) | NR | NR | NR / 25.4 ± 3.7 | NR / 26.9 ± 4.9 |
| Chowdhury (2010) | 41.10±1.18 | 42.80±1.59 | 19 (48.71%) | 21(52.67%) | NR | NR | 57.20±1.77 / NR | 56.80±2.36 / NR |
| Hussain (2015) | 37.70±6.58 | 37.53±6.71 | 12(40.00%) | 14(46.67%) | NR | NR | NR/NR | NR/NR |
| Jellish(1996) | 43±2 | 46±2 | 26 | 30 | NR | NR | 84.3±2.4 / NR | 84.9±2.3/ NR |
| Kahveci (2014) | 48.10±12.17 | 47.95±11.6 | 12 | 18 | 19/21/0 | 22/18/0 | 75.75±9.44 / NR | 76.35±7.72 / NR |
| Kara (2011) | 51+-NR | 49+-NR | 14 (46.67%) | 13(43.33%) | 16/14/0 | 19/11/0 | NR / NR | NR / NR |
| Kilic (2019) | 54.1 ± 2.4 | 53.2 ± 3.1 | 24 (48%) | 26 (52%) | 18/30/2 | 20/28/2** | NR / 27.33 ± 3.69 | NR / 29.15 ± 5.37 |
| Sadrolsadat (2009) | 45.7 ± 5.2 | 45.2 ± 5.6 | NR | NR | NR | NR | 77.8 ± 7.5/ NR | 75.2 ± 8.2/ NR |
| Vural (2014) | NR | Nr | NR | NR | NR | NR | NR / NR | NR / NR |
| Yildirim Güçlü (2014) | NR | NR | NR | NR | NR | NR | NR / NR | NR / NR |

*Data were expressed as mean*±*standard deviation, median and interquartile range or as number and (%). SA: Spinal Anesthesia; GA: General Anesthesia; F: females; IQR: Interquartile Range: NR: not reported,*

**Secondary outcomes timings**

| Study | PO Analgesics | Nausea/Vomiting | Urinary retention | Surgeon Satisfaction | Patient Satisfaction |
| --- | --- | --- | --- | --- | --- |
| Attari (2011) | First 24 hours after surgery | First 24 hours after surgery | - | - | Satisfaction after surgery |
| Baenziger (2020) | - | First 24 hours after surgery | In PACU | - | At discharge |
| Chowdhury (2010) | - | Postoperative | Postoperative | - | Comfort after surgery |
| Hussain (2015) | - | First 24 hours after surgery | First 24 hours after surgery | - | - |
| Jellish (1996) | PACU | First 24 hours after surgery | First 24 hours after surgery | - | - |
| Kahveci (2014) | PACU | PACU | - | Related to anesthesia | - |
| Kara (2011) | PACU | First 24 hours after surgery | First 24 hours after surgery | Related to anesthesia | Comfort during anesthesia |
| Kilic (2019) | PACU | - | - | - | At discharge |
| Sadrolsadat (2009) | PACU | First 24 hours after surgery | - | Related to anesthesia |  |
| Vural (2014) | - | Postoperative | Postoperative | - | At discharge |
| Yildirim Güçlü (2014) | - | First 24 hours after surgery | First 24 hours after surgery | - | - |
