# Supplementary material for: General Anesthesia Compared to Spinal Anesthesia for Patients Undergoing Lumbar Vertebral Surgery: A Meta-Analysis of Randomized Controlled Trials
Source: J Clin Med. 2020 Dec 30;10(1):102. doi: 10.3390/jcm10010102 (PMC7796239; doi:10.3390/jcm10010102)
Supplement: Supplementary file 1 [file jcm-10-00102-s001.zip › Suppl/Table S3.docx]

Table S3. Bias assessment based on Attari (2011).

| **Entry** | **Judgment** | **Description** |
| --- | --- | --- |
| **Randomization** | *Low risk* | Allocation sequence was concealed (sealed envelope) but there is no information on how the sequence was generated.No imbalances are apparent. |
| **Deviations** | *Low risk* | Both participants and personnel were aware of the intervention, however there were no described deviations because of the trial context. The analysis used to estimate the effect of assignment to intervention was appropriated. |
| **Missing outcome** | *Low risk* | There were data for the analyzed outcomes available for all participants randomized. |
| **Measurement** | *Some concerns* | The method for measuring the outcomes was appropriated and was not different among the groups. The outcome assessor was probably aware of the intervention. Knowledge of intervention status could have  influenced outcome assessment but there is no reason to believe that it did. |
| **Selection** | *Some concerns* | Analysis intentions are not available. |
| **Overall** | *Some concerns* | The study is judged to raise some concerns in at least one domain for this result, but not to be at high risk of bias for any domain. |

**Baenziger (2020)**

| **Entry** | **Judgment** | **Description** |
| --- | --- | --- |
| **Randomization** | *Low risk* | The allocation sequence was generated by an independent statistician and based on computer generated random numbers. No imbalances are apparent |
| **Deviations** | *Low risk* | Both participants and personnel were aware of the intervention, however there were no described deviations because of the trial context. The analysis used to estimate the effect of assignment to intervention was appropriated |
| **Missing outcome** | *Low risk* | There were data for the analyzed outcomes available for all participants randomized |
| **Measurement** | *Low risk* | The method for measuring the outcomes was appropriated and was not different among the groups. The outcome assessor was not aware of the intervention. |
| **Selection** | *Some concerns* | Analysis intentions were available but several different outcomes were analyzed (NCT03300089). |
| **Overall** | *Some concerns* | The study is judged to raise some concerns in at least one domain for this result, but not to be at high risk of bias for any domain. |

**Chowdhury(2010)**

| **Entry** | **Judgment** | **Description** |
| --- | --- | --- |
| **Randomization** | *Some concerns* | There is no information on allocation concealment but no baseline imbalances are apparent |
| **Deviations** | *Low risk* | Both participants and personnel were aware of the intervention, however there were no described deviations because of the trial context. The analysis used to estimate the effect of assignment to intervention was appropriated |
| **Missing outcome** | *Low risk* | There were data for the analyzed outcomes available for all participants randomized |
| **Measurement** | *Some concerns* | The method for measuring the outcomes was appropriated and was not different among the groups. The outcome assessor was probably aware of the intervention. Knowledge of intervention status could have  influenced outcome assessment but there is no reason to believe that it did. |
| **Selection** | *Some concerns* | Analysis intentions are not available. |
| **Overall** | *Some concerns* | The study is judged to raise some concerns in at least one domain for this result, but not to be at high risk of bias for any domain. |

**Hussain (2015)**

| **Entry** | **Judgment** | **Description** |
| --- | --- | --- |
| **Randomization** | *Some concerns* | There is no information on allocation concealment but no baseline imbalances are apparent |
| **Deviations** | *Low risk* | Both participants and personnel were aware of the intervention, however there were no described deviations because of the trial context. The analysis used to estimate the effect of assignment to intervention was appropriated |
| **Missing outcome** | *Low risk* | There were data for the analyzed outcomes available for all participants randomized |
| **Measurement** | *Some concerns* | The method for measuring the outcomes was appropriated and was not different among the groups. The outcome assessor was probably aware of the intervention. Knowledge of intervention status could have  influenced outcome assessment but there is no reason to believe that it did. |
| **Selection** | *Some concerns* | Analysis intentions are not available. |
| **Overall** | *Some concerns* | The study is judged to raise some concerns in at least one domain for this result, but not to be at high risk of bias for any domain. |

**Jellish (1996)**

| **Entry** | **Judgment** | **Description** |
| --- | --- | --- |
| **Randomization** | *Some concerns* | There is no information on allocation concealment but no baseline imbalances are apparent |
| **Deviations** | *Low risk* | Both participants and personnel were aware of the intervention, however there were no described deviations because of the trial context. The analysis used to estimate the effect of assignment to intervention was appropriated |
| **Missing outcome** | *Low risk* | There were data for the analyzed outcomes available for all participants randomized |
| **Measurement** | *Some concerns* | The method for measuring the outcomes was appropriated and was not different among the groups. The outcome assessor was probably aware of the intervention. Knowledge of intervention status could have  influenced outcome assessment but there is no reason to believe that it did. |
| **Selection** | *Some concerns* | Analysis intentions are not available. |
| **Overall** | *Some concerns* | The study is judged to raise some concerns in at least one domain for this result, but not to be at high risk of bias for any domain. |

**Kahveci (2014)**

| **Entry** | **Judgment** | **Description** |
| --- | --- | --- |
| **Randomization** | *Some concerns* | There is no information on allocation concealment but no baseline imbalances are apparent |
| **Deviations** | *Low risk* | Both participants and personnel were aware of the intervention, however there were no described deviations because of the trial context. The analysis used to estimate the effect of assignment to intervention was appropriated |
| **Missing outcome** | *Low risk* | There were data for the analyzed outcomes available for all participants randomized |
| **Measurement** | *Some concerns* | The method for measuring the outcomes was appropriated and was not different among the groups. The outcome assessor was probably aware of the intervention. Knowledge of intervention status could have  influenced outcome assessment but there is no reason to believe that it did. |
| **Selection** | *Some concerns* | Analysis intentions are not available. |
| **Overall** | *Some concerns* | The study is judged to raise some concerns in at least one domain for this result, but not to be at high risk of bias for any domain. |

**Kara (2011)**

| **Entry** | **Judgment** | **Description** |
| --- | --- | --- |
| **Randomization** | *Some concerns* | There is no information on allocation concealment but no baseline imbalances are apparent |
| **Deviations** | *Low risk* | Both participants and personnel were aware of the intervention, however there were no described deviations because of the trial context. The analysis used to estimate the effect of assignment to intervention was appropriated |
| **Missing outcome** | *Low risk* | There were data for the analyzed outcomes available for all participants randomized |
| **Measurement** | *Some concerns* | The method for measuring the outcomes was appropriated and was not different among the groups. The outcome assessor was probably aware of the intervention. Knowledge of intervention status could have  influenced outcome assessment but there is no reason to believe that it did. |
| **Selection** | *Some concerns* | Analysis intentions are not available. |
| **Overall** | *Some concerns* | The study is judged to raise some concerns in at least one domain for this result, but not to be at high risk of bias for any domain. |

**Kilic (2019)**

| **Entry** | **Judgment** | **Description** |
| --- | --- | --- |
| **Randomization** | *High risk* | Group assignment was based on groups based  on the identification card first number. Allocation was probably not concealed. |
| **Deviations** | *Low risk* | Both participants and personnel were aware of the intervention, however there were no described deviations because of the trial context. The analysis used to estimate the effect of assignment to intervention was appropriated |
| **Missing outcome** | *Low risk* | There were data for the analyzed outcomes available for all participants randomized |
| **Measurement** | *Some concern* | The method for measuring the outcomes was appropriated and was not different among the groups. The outcome assessor was probably aware of the intervention. Knowledge of intervention status could have  influenced outcome assessment but there is no reason to believe that it did. |
| **Selection** | *Some concerns* | Analysis intentions are not available. |
| **Overall** | *High risk* | The study is judged to be at high risk of bias in one domain |

**Sadrolsadat (2009)**

| **Entry** | **Judgment** | **Description** |
| --- | --- | --- |
| **Randomization** | *Some concerns* | There is no information on allocation concealment but no baseline imbalances are apparent |
| **Deviations** | *Low risk* | Both participants and personnel were aware of the intervention, however there were no described deviations because of the trial context. The analysis used to estimate the effect of assignment to intervention was appropriated |
| **Missing outcome** | *Low risk* | There were data for the analyzed outcomes available for all participants randomized |
| **Measurement** | *Low risk* | The method for measuring the outcomes was appropriated and was not different among the groups. The outcome assessor was a trained technician unaware of the objectives of the study |
| **Selection** | *Some concerns* | Analysis intentions are not available. |
| **Overall** | *Some concerns* | The study is judged to raise some concerns in at least one domain for this result, but not to be at high risk of bias for any domain. |

**Vural (2013)**

| **Entry** | **Judgment** | **Description** |
| --- | --- | --- |
| **Randomization** | *Some concerns* | There is no information on both allocation concealment and randomization method used but no baseline imbalances are apparent |
| **Deviations** | *Low risk* | Both participants and personnel were aware of the intervention, however there were no described deviations because of the trial context. The analysis used to estimate the effect of assignment to intervention was appropriated |
| **Missing outcome** | *Low risk* | There were data for the analyzed outcomes available for all participants randomized |
| **Measurement** | *Some concerns* | The method for measuring the outcomes was appropriated and was not different among the groups. The outcome assessor was probably aware of the intervention. Knowledge of intervention status could have  influenced outcome assessment but there is no reason to believe that it did. |
| **Selection** | *Some concerns* | Analysis intentions are not available. |
| **Overall** | *Some concerns* | The study is judged to raise some concerns in at least one domain for this result, but not to be at high risk of bias for any domain. |

**Yildirim Güçlü (2014)**

| **Entry** | **Judgment** | **Description** |
| --- | --- | --- |
| **Randomization** | *Low risk* | Allocation sequence was concealed (sealed envelope) but there is no information on how the sequence was generated.No imbalances are apparent |
| **Deviations** | *Low risk* | Both participants and personnel were aware of the intervention, however there were no described deviations because of the trial context. The analysis used to estimate the effect of assignment to intervention was appropriated |
| **Missing outcome** | *Low risk* | There were data for the analyzed outcomes available for all participants randomized |
| **Measurement** | *Some concerns* | The method for measuring the outcomes was appropriated and was not different among the groups. The outcome assessor was probably aware of the intervention. Knowledge of intervention status could have  influenced outcome assessment but there is no reason to believe that it did. |
| **Selection** | *Some concerns* | Analysis intentions are not available. |
| **Overall** | *Some concerns* | The study is judged to raise some concerns in at least one domain for this result, but not to be at high risk of bias for any domain. |
