# Supplementary material for: General Anesthesia Compared to Spinal Anesthesia for Patients Undergoing Lumbar Vertebral Surgery: A Meta-Analysis of Randomized Controlled Trials
Source: J Clin Med. 2020 Dec 30;10(1):102. doi: 10.3390/jcm10010102 (PMC7796239; doi:10.3390/jcm10010102)
Supplement: Supplementary file 1 [file jcm-10-00102-s001.zip › Suppl/Table S4.docx]

| Table S4. Summary of findings table. | | | | | | | | | | |
| --- | --- | --- | --- | --- | --- | --- | --- | --- | --- | --- |
| **Certainty assessment** | | | | | | | **Summary of findings** | | | |
| **Participants  (studies) Follow up** | **Risk of bias** | **Inconsistency** | **Indirectness** | **Imprecision** | **Publication bias** | **Overall certainty of evidence** | **Study event rates (%)** | | **Relative effect (95% CI)** | **Risk difference with General** |
|  |  |  |  |  |  |  | **With General** | **With Spinal** |  |  |
| **Postoperative pain** | | | | | | | | | | |
| 488 (6 RCTs) | serious ^a^ | very serious ^i^ | not serious | not serious | none | ⨁◯◯◯ VERY LOW | 244 | 244 | - | SMD **2.32 higher** (3.91 higher to 0.73 higher) |
| **Analgesic Requirement** | | | | | | | | | | |
| 534 (6 RCTs) | serious ^b^ | serious ^j^ | not serious | not serious | none | ⨁⨁◯◯ LOW | 170/268 (63.4%) | 46/266 (17.3%) | **OR 11.52** (5.12 to 25.93) | **534 more per 1.000** (from 344 fewer to 671 more) |
| **Blood Loss** | | | | | | | | | | |
| 554 (6 RCTs) | serious ^b^ | very serious ^d^ | not serious | not serious | none | ⨁◯◯◯ VERY LOW | 268 | 286 | - | MD **53.88 higher** (98.13 higher to 9.63 higher) |
| **Surgery Length** | | | | | | | | | | |
| 750 (9 RCTs) | serious ^e^ | very serious ^f^ | not serious | not serious | none | ⨁◯◯◯ VERY LOW | 376 | 374 | - | MD **4.56 higher** (13.16 higher to 4.04 lower) |
| **Hypotension** | | | | | | | | | | |
| 580 (7 RCTs) | serious ^a^ | serious ^g^ | not serious | not serious | none | ⨁⨁◯◯ LOW | 60/291 (20.6%) | 88/289 (30.4%) | **OR 0.51** (0.23 to 1.11) | **23 fewer per 1.000** (from 45 fewer to 6 fewer) |
| **Bradycardia** | | | | | | | | | | |
| 520 (6 RCTs) | serious ^a^ | serious ^h^ | not serious | not serious | none | ⨁⨁◯◯ LOW | 38/261 (14.6%) | 45/259 (17.4%) | **OR 0.74** (0.30 to 1.80) | **34 fewer per 1.000** (from 97 fewer to 89 more) |
| **Nausea and vomiting** | | | | | | | | | | |
| 796 (10 RCTs) | serious ^a^ | not serious | not serious | not serious | none | ⨁⨁⨁◯ MODERATE | 73/399 (18.2%) | 30/397 (7.5%) | **OR 2.69** (1.73 to 4.20) | **187 more per 1.000** (from 91 more to 296 more) |
| **Urinary retention** | | | | | | | | | | |
| 544 (7 RCTs) | serious ^a^ | not serious | not serious | not serious | none | ⨁⨁⨁◯ MODERATE | 31/272 (11.8%) | 35/272 (12.9%) | **OR 1.15** (0.68 to 1.94) | **26 more per 1.000** (from 32 fewer to 109 more) |
| **Length of Stay** | | | | | | | | | | |
| 578 (7 RCTs) | serious ^b^ | serious ^c^ | not serious | not serious | none | ⨁⨁◯◯ LOW | 289 | 289 | - | MD **0.31 higher** (0.41 higher to 0.21 higher) |
| **Patient Satisfaction** | | | | | | | | | | |
| 478 (6 RCTs) | serious ^a^ | not serious | not serious | not serious | none | ⨁⨁⨁◯ MODERATE | 207/240 (86.3%) | 227/238 (95.3%) | **OR 0.38** (0.12 to 1.16) | **85 fewer per 1.000** (from 186 fewer to 25 fewer) |
| **Surgeon Satisfaction** | | | | | | | | | | |
| 240 (3 RCTs) | serious ^b^ | serious ^d^ | not serious | not serious | none | ⨁⨁◯◯ LOW | 120 | 120 | - | MD **11.08 higher** (13.56 lower to 8.6 lower) |

**CI:** Confidence interval; **OR:** Odds ratio; **MD:** Mean difference; **RR:** Risk ratio; **SMD:** Standardised mean difference.

a. Many studies are at moderate risk of bias; b. Many studies are at moderate risk of bias and one is at high risk; c. I2=54%, p <0.001; d. I2=97%, p=0.02; e. Many studies are at moderate risk of bias, one study at high risk and one at high risk; f. I2=98%, p=0.03; g. I2=61%, p=0.09; h. I2=55%, p=0.51; i. I2=98%, p=0.004, j. I^2^ 56%, p 0.001.
